# A novel acquired inv(2)(p23.3q24.3) with concurrent submicroscopic deletions at 2p23.3, 2p22.1, 2q24.3 and 1p13.2 in a patient with chronic thrombocytopenia and anemia

**DOI:** 10.1186/s13039-015-0113-z

**Published:** 2015-02-01

**Authors:** Eigil Kjeldsen

**Affiliations:** HemoDiagnostic Laboratory, CancerCytogenetic Section, Department of Hematology, Aarhus University Hospital, Tage-Hansens Gade 2, Ent. 4A, DK-8000 Aarhus C, Denmark

**Keywords:** Thrombocytopenia, Anemia, Chromosome 2, Inversion, Microdeletion, aCGH

## Abstract

**Background:**

Thrombocytopenia can result from a wide range of conditions and may be determined by multiple mechanisms. It can be due to a reduced platelet production or an increased destruction of platelets. Increased destruction is seen in conditions such as disseminated intravascular coagulation (DIC) and thrombotic microangiopathies, whereas decreased production is seen in bone marrow (BM) failure syndromes such as aplastic anemia, myelodysplastic syndromes, and chemotherapy-induced thrombocytopenia. In BM failure syndromes thrombocytopenia is often accompanied by anemia and/or leucopenia. Recognition of the cause of thrombocytopenia is often crucial for correct management of patients.

**Case presentation:**

Here, we report on a 71 year-old male caucasian with thrombocytopenia since six years, and a recent development of anemia. At the time of progression with anemia a bone marrow sampling was done to examine for a possible causative myeloid malignancy. The morphological examination was normal whereas immunophenotyping by flowcytometry could not exclude myelodysplasia. Cytogenetic analysis by G-banding revealed a pericentric inversion of chromosome 2 in 23 out of 25 analyzed metaphases. The inversion was further characterized by molecular cytogenetics and high-resolution oligo-based array-CGH analysis. Together the analyses demonstrated a 141.8 Mb pericentric inversion, inv(2)(p23.3q24.3), and concurrent submicroscopic deletions in 2p23.3, 2p22.1, 2q24.3 and 1p13.2 between 0.6-1.9 Mb in size. Locus-specific FISH analyses confirmed all deletions and the pericentric inversion of chromosome 2. The chromosomal abnormalities were present in 87% of the bone marrow cells whereas analysis of a skin biopsy revealed a normal male karyotype as well as a normal array-CGH result. These findings demonstrate that the identified abnormalities were acquired.

**Conclusion:**

To the best of our knowledge, this is the first report of chronic thrombocytopenia and anemia associated with acquired inv(2)(p23.3q24.3) as a sole cytogenetic abnormality together with concurrent submicroscopic deletions at 2p23.3, 2p22.1, 2q24.3 and 1p13.2.

**Electronic supplementary material:**

The online version of this article (doi:10.1186/s13039-015-0113-z) contains supplementary material, which is available to authorized users.

## Background

Idiopathic thrombocytopenic purpura (ITP) is a common hematologic disorder that affects patients of all ages and with equal sex ratio [1,2]. Primary ITP is defined as isolated thrombocytopenia (platelet count < 100 ×10^9^/L) in absence of other causes or disorders that may be associated with thrombocytopenia. Chronic thrombocytopenia is defined as disease of more than 12 months duration. Acquired thrombocytopenia may also be part of pancytopenia in myelodysplastic syndromes (MDS) or in acute myeloid leukemia (AML) when either anemia and/or leucopenia are present [3].

Recognition of the cause of thrombocytopenia is important for proper treatment of the patients. Cytogenetic evaluation is a prerequisite in diagnostic work-up of myeloid malignancies to provide valuable prognostic information and to determine treatment strategies [4]. Several recurrent cytogenetic abnormalities have been identified in myeloid malignancies and these have conveyed into risk stratification groups [5-8].

In cases with isolated thrombocytopenia cytogenetic evaluation is uncommon and only few cytogenetic abnormalities have been associated with isolated thrombocytopenia. Thrombocytopenia-absent radius syndrome is a rare autosomal recessive disorder that has been associated with either *RBM8A* gene mutation or a 1.35 Mb microdeletion in 1q21.1 [9,10]. In cases where isolated thrombocytopenia evolves with additional cytopenias or in cases where these are present upfront it is suggestive of possible underlying MDS or AML [3]. In these cases examination of the bone marrow by morphology, flow cytometry and cytogenetic analyses are usually performed.

Here is reported the molecular cytogenetic and array-based CGH characterization of a patient with chronic thrombocytopenia and anemia without dysplastic signs or increased blasts in the bone marrow. We identified a novel acquired pericentric inversion of chromosome 2, inv(2)(p23.3q24.3), as a sole cytogenetic abnormality together with four additional concurrent submicroscopic deletions in 2p23.3, 2p22.1, and 2q24.3 and 1p13.2. These chromosomal abnormalities have not been reported previously.

## Case presentation

### Clinical report

A 71-year old male was referred to our Hematological Department in January 2013 suspected for MDS. He had an acute myocardial infarction (AMI) in 1999 and recovered from this without complications except for dyspnea. He has been on anti-thrombotic medication with Warfarin and Aspirin since. At the time of referral to our department he had had thrombocytopenia since 2008 with values between 87–124 × 10^9^/L and developed additionally moderate anemia (hemoglobin 7.4 mM) in December 2012. His white blood cell counts were in the years 2008–2013 within normal range whereas he had a moderate increase in LDH with values between 264–398 U/L. Physical examination revealed areas with bruising of his skin. He has epistaxis once every second month. He had been smoking until approximately 6 months prior to referral with a calculated 10 pack years.

As part of the diagnostic work-up in January 2013 a bone marrow examination was performed including morphology and cytogenetic analyses. The morphological examination was normal whereas the cytogenetics analysis revealed a pericentric inversion of chromosome 2 in 23 out of 25 analyzed metaphases. Watchful waiting was decided with additional cytogenetic work-up (se below for further description). Eighteen month after referral a follow-up examination of his bone marrow cells included morphology, flow cytometry and cytogenetic analyses. Morphology examination was still without dysplastic signs or increased blasts whereas flow cytometry could not exclude myelodysplasia. Biochemistry at one-year follow-up was without progression and showed continued thrombocytopenia (platelets, 92 × 10^9^/L) with moderate anemia (hemoglobin, 7.2 mM). He is continuously well and without development in symptoms, and visits our hospital for regular follow-ups.

## Methods

### G-banding analysis

G-banded chromosomes were prepared after short-term unstimulated culturing of bone marrow cells, and PHA-stimulated peripheral blood cells as described [11]. Karyotypes were described according to [12].

### Fluorescent in situ hybridization (FISH) analysis

Human multicolor FISH were done according to manufacturer’s instructions using the following XCyting multicolor FISH probes: 1) 24-color karyotyping was done with the 24Xyte kit consisting of 24 different chromosome painting probes; and 2) mBanding with XCyte 2 probes consisting of a series of partial chromosome paint probes for sequential partially overlapping chromosome regions of a single chromosome (MetaSystems, Altlussheim, Germany). Each of the XCyte probes was labeled with one of five fluorochromes or a unique combination thereof (combinatorial labeling). Metaphases were counterstained with 4’,6-diamidino-2-phenylindole (DAPI). Image capture was done with an automated Zeiss Axio Imager.Z2 equipped with a CCD-camera (CoolCube1) and appropriate filters using Isis software (MetaSystems). Karyotyping was done using the 24-color mFISH upgrade package, ISIS, including mBanding.

Whole chromosome painting, chromosome arms specific painting and locus specific analysis was done with the following directly labeled probes according to manufacturer’s instructions: 1) whole chromosome painting probes for chromosome 2 (Kreatech Diagnostics, Amsterdam, The Netherlands); 2) arms specific painting probes for chromosome arms 2p and 2q (Kreatech Diagnostics); 3) LSI ALK dual color, break apart rearrangement probe (Abbott Molecular, Wiesbaden, Germany); and 4) SE1 (1qh), satellite III probe (Kreatech). Table 1 summarizes the custom made BAC-based probes (Empire Genomics, New York, USA) for validating the oaCGH findings. Chromosomes were counterstained with DAPI.

**Table 1:** **Summary of custom made BAC-based probes for characterization and validation of oaCGH findings**

**BAC probe**	**Cytoband**	**Genomic position (bp)** ^**a**^
RP11-349M4	1p13.2	114,355,404-114,540,804
RP11-89G5	2p23.3	25,523,249-25,692,080
RP11-730G3	2p23.3	25,833,964-26,035,145
RP11-106P6	2p22.1	39,558,258-39,718,842
RP11-1104H5	2q24.3	169,206,762-169,415,852
RP11-937M17	2q31.1	169,560,846-169,737,939

^a^Genomic positions are given according to NCBI build 36.1 (hg18).

### Oligo-based array comparative genomic hybridization (oaCGH) analysis

oaCGH analysis was performed using CytoChip Cancer 4×180K v2.0 (BlueGnome, Cambridge, UK) encompassing a 20 kb backbone with highest concentration of probes at 670 cancer genes. The analysis was done according to manufacturer’s instructions using 0.5 μg patient DNA from bone marrow cells at initial diagnosis as described in [11]. After hybridization, washing and drying the oligo array was scanned at 2.5 μm with GenePix 4400A microarray scanner. Initial analysis and normalization was done with BlueFuseMulti v2.6. For analysis and visualization normalized log2 probe signal values were imported into Nexus Copy Number software v. 6.1 (BioDiscovery, California, USA) and segmented using FASST2 segmentation algorithm with a minimum of 3 probes/segment. Regions of gain or loss contained within copy number variable regions (CNVs) were discarded. Reference genome was NCBI build 36.1 (hg18). Bioinformatics analysis was performed by querying the UCSC database (http://genome.ucsc.edu).

## Results

### G-banding and molecular cytogenetic analysis

**Figure 1:**
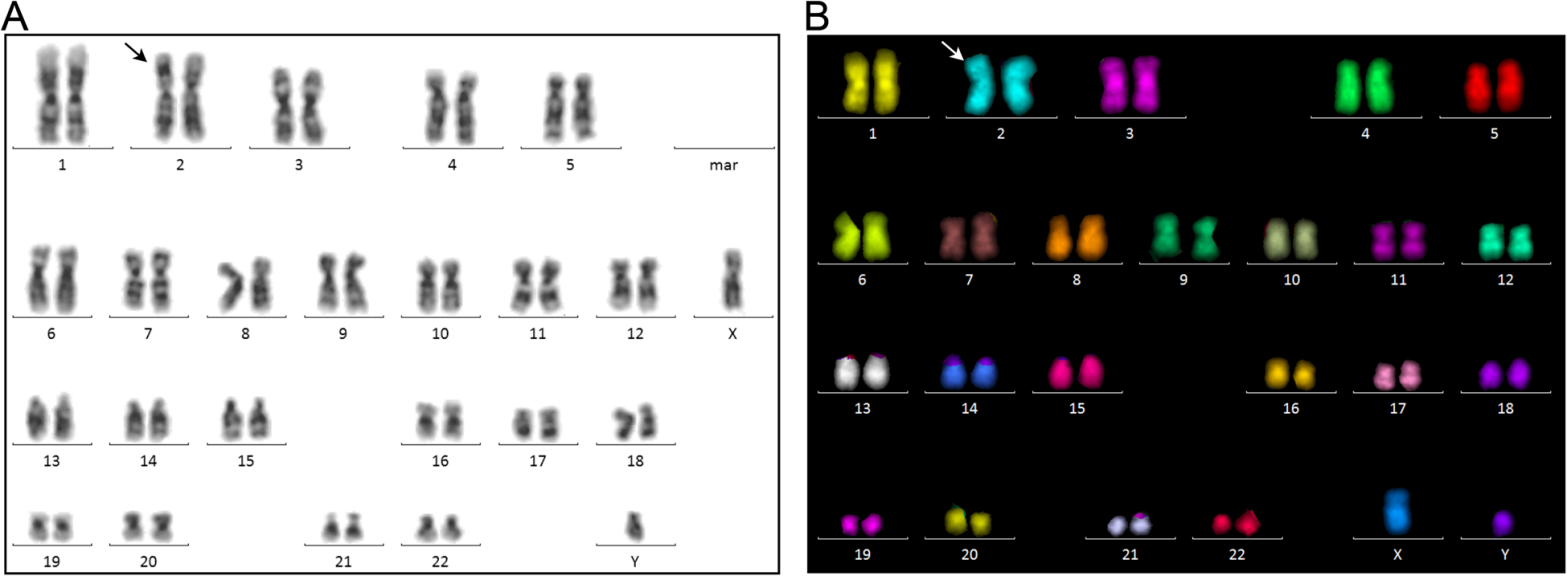
**Karyotyping analyses.** Panel **A**. G-banding analysis showed an aberrant karyotype initially interpreted as 46,XY,inv(2)(p22q31)[23]/46,XX[2]. Panel **B**. 24-color karyotyping did not reveal any additional cryptic rearrangement. Arrows indicate the aberrant chromosome 2.

**Figure 2:**
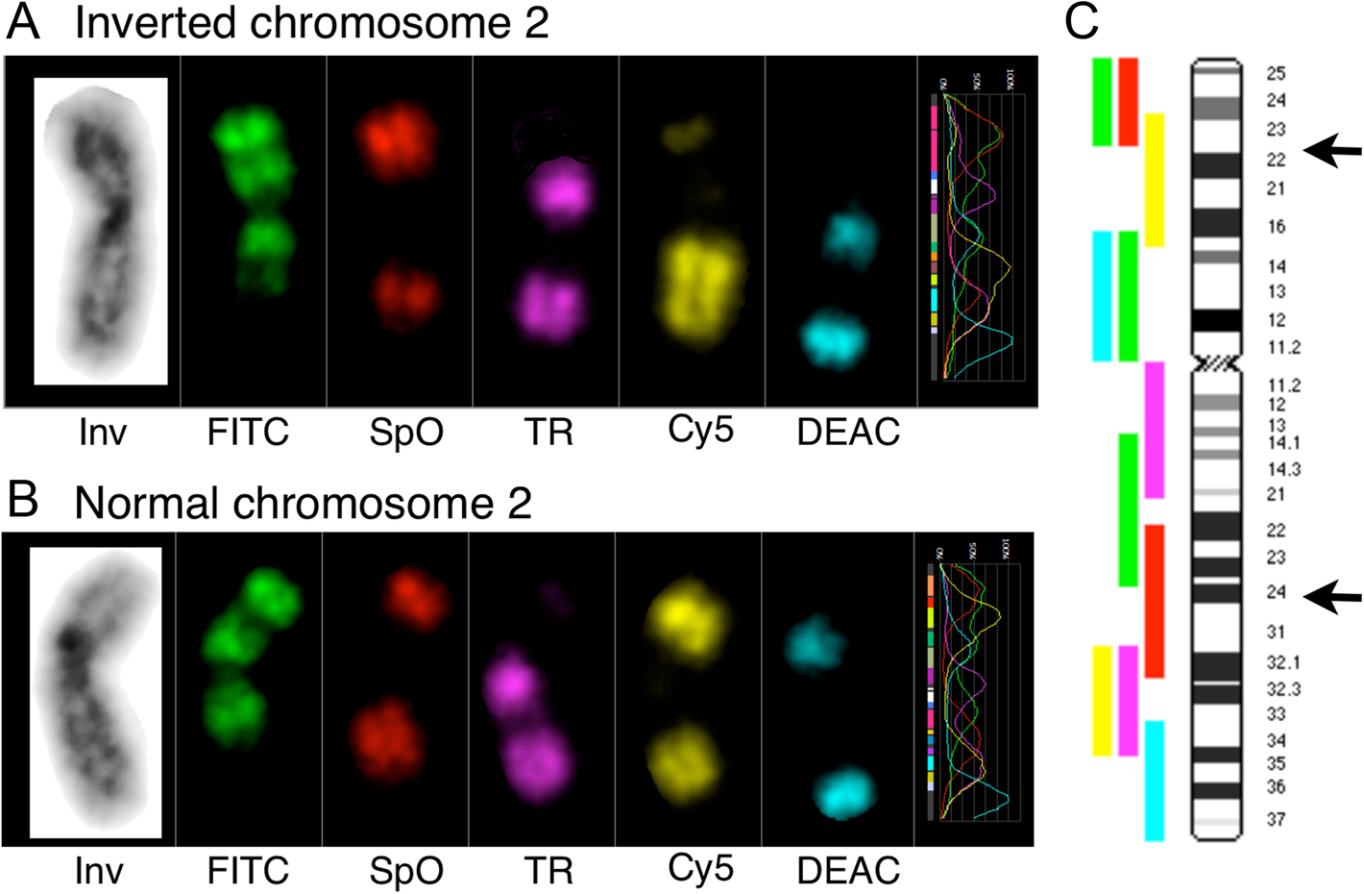
**mBanding analysis of chromosome 2.** The single color gallery tool in ISIS software shows individual color schemes of labeled chromosomes arranged in their capture sequence (fluorescein isothiocyanate) FITC, (spectrum orange) SpO, (texas red) TR, (cyanine 5) Cy5, (7-diethylaminocoumarin-3-carboxylic acid, succinimidyl ester) DEAC, together with an inverted grayscale image of the DAPI image (Inv). Panel **A**. Inverted chromosome 2. Panel **B**. A normal chromosome 2 from the patient’s karyotype. Panel **C**. A schematic representation of the localization of the different multicolor probes of XCyte 2 relative to the ideogram of chromosome 2 together with breakpoints marked by arrows.

Karyotyping by G-banding of unstimulated bone marrow cells at the time of referral was interpreted as an apparently balanced male karyotype 46,XY, inv(2)(p22q31)[23]/46,XY[2] showing a predominant pericentric inversion of chromosome 2 (Figure 1A). No additional cryptic aberrations were revealed after 24-color karyotyping using the 24XCyte human multicolor (mFISH) probe kit (Figure 1B). To more precisely define which chromosomal band regions on chromosome 2 that are involved in the inversion we performed mBanding with the mBand probe kit XCyte 2. This analysis confirmed the pericentric inversion and a minor revision of the karyotype 46,XY, inv(2)(p23q24)[23]/46,XY[2] could be made (Figure 2). Analysis of phytohemagglutinin stimulated cultures of blood lymphocytes also showed the pericentric inversion of chromosome 2 but with less abnormal metaphases as indicated in the karyotype 46,XY, inv(2)(p23q24)[2]/46,XY[18]. From this result we could not determine whether the inv(2) was a constitutional abnormality. For this reason it was decided to perform cytogenetic analysis of a skin biopsy, which was done six months later at the local Department of Clinical Genetics, and revealed a normal male karyotype. Cytogenetic analysis of his bone marrow cells at eighteen months follow-up showed an unchanged abnormal karyotype.

### Microarray analysis

**Figure 3:**
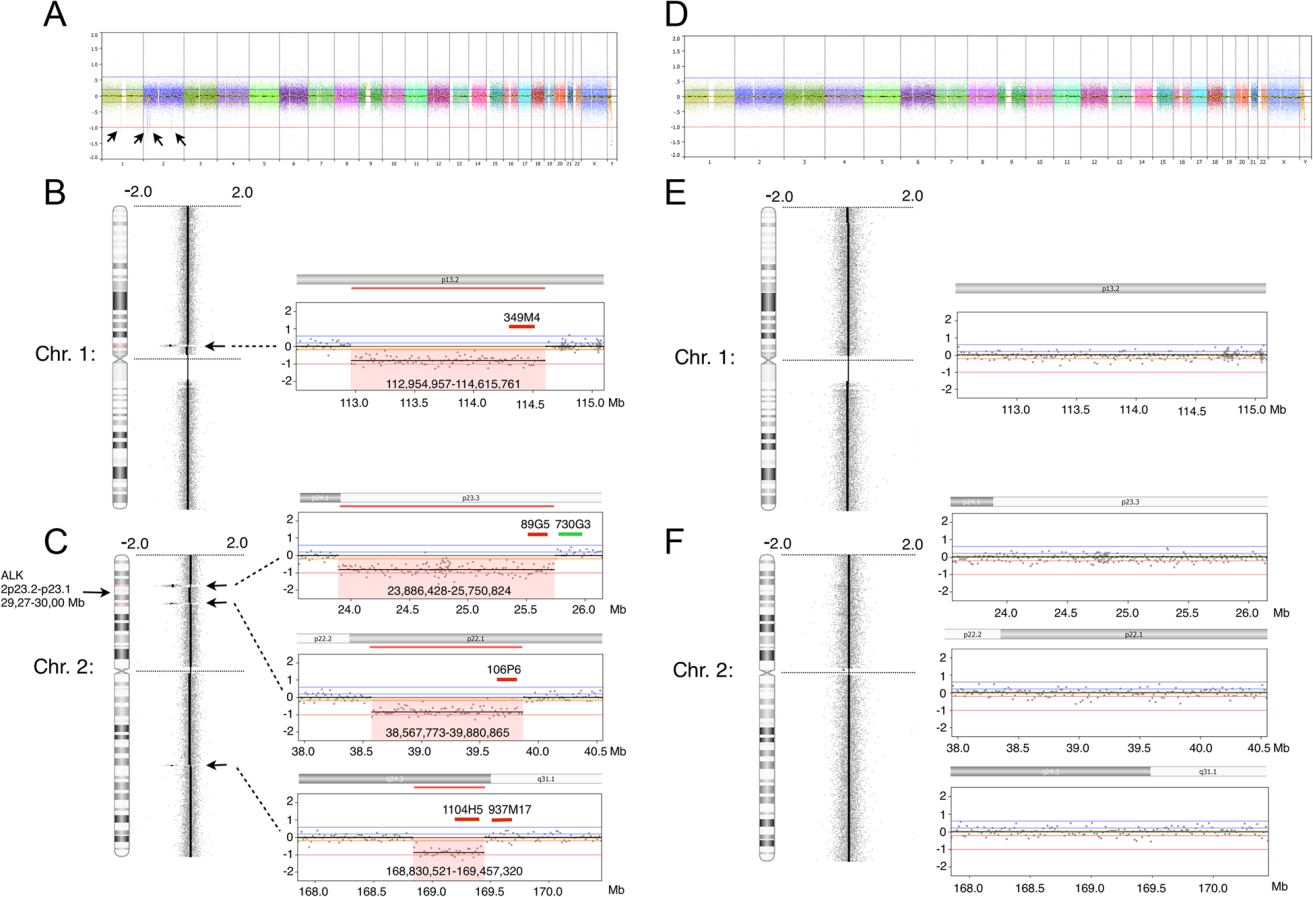
**Genome analysis using high resolution 180 K oligo-based array CGH analysis.** Panels **A**-**C**. aCGH analysis of DNA from bone marrow at the time of referral. Panels **D**-**F**. aCGH analysis of DNA from a skin biopsy. Vertical blue lines indicate log_2_ ratios +0.24 and +0.60 and red lines indicate log_2_ ratios −0.24 and −1.0. The X-axis at the bottom indicates chromosomal position. Panel **A**. Whole genome view of the bone marrow sample showing a total of four submicroscopic deletions at chromosomes 1 and 2 indicated by arrows. Panel **B**. To the left a chromosome view of chromosome 1 with deletion at 1p13.2 indicated by an arrow. To the right a zoom view of the deleted region as indicated by red shade together with its maximal chromosomal position. Panel **C**. To the left the chromosome view of chromosome 2 with deletions at 2p23.3, 2p22.1 and 2q24.3 as indicated by arrows. To the right zoom views of the deleted regions as indicated by red shade together with their respective maximal chromosomal positions. Panel **D**. Whole genome view of skin biopsy sample lacking the submicroscopic deletions. Panel **E**. To the left a chromosome view of chromosome 1 and to the right a zoom view of the same region as in Panel **B**. Panel **F**. To the left a chromosome view of chromosome 2 and to the right a zoom view of the same regions as in Panel **C**. The relative positions of the different FISH probes used for validation are indicated in different colors according to the direct fluorescent label used.

To search for possible copy number abnormalities associated with the pericentric inv(2) we performed oaCGH analysis using the CytoChip Cancer 4x180K v2.0 (BlueGnome, Cambridge, UK). We used purified DNA from the bone marrow cells and pooled male DNA as reference. The oaCGH analysis detected the following copy number alterations: one microdeletion at 1p13.2 and three microdeletions at 2p23.3, 2p22.1 and 2q24.3 (Figure 3A-C). The microdeletion in chromosome band 1p13.2 has of maximal size 1,660 kb (pos. 112,954,957-114,615,761; encompassing the oligonucleotide probes A_14_P134475 to A_16_P152654439) and a minimum size of 1,624 kb (pos. 112,969,151-114,593,882; encompassing the oligo-nucleotide probes A_16_P35272698 to A_16_P15265386). The maximum regions of deletions involved in the other break point regions are: 1) at chromosome band 2p23.3 the microdeletion encompasses the oligo-nucleotide probes A_16_P15585250 to A_16_P00335733 mapping from 23,886,428 to 25,750,824; 2) at chromosome band 2p22.1 the microdeletion encompasses the oligo-nucleotide probes A_16_P00353846 to A_16_P15623187 mapping from 38,567,773 to 39,880,865; and 3) at chromosome band 2q24.3 the microdeletion encompasses the oligo-nucleotide probes A_16_P35951089 to A_16_P35952722 mapping from 168,830,521 to 169,457,320. The minimum region of microdeletions involved in the breakpoints are: 1) at chromosome band 2p23.3 the microdeletion encompasses the oligo-nucleotide probes A_16_P15585279 to A_16_P00335710 mapping from 23,897,035 to 25,729,092; 2) at chromosome band 2p22.1 the microdeletion encompasses the oligo-nucleotide probes A_16_P15619997 to A_16_P35646932 mapping from 38,576,675 to 39,859,229; and 3) at chromosome band 2q24.3 the microdeletion encompasses the oligo-nucleotide probes A_16_P00523728 to A_14_P101380 mapping from 168,846,741 to 169,438,394. From these results the respective estimated minimum to maximum deletion sizes are: 1) at 2p23.3: 1,831-1,864 kb; 2) at 2p22.1: 1,280-1,313 kb; and 3) at 2q24.3: 591–626 kb.

To examine whether the observed microdeletions were constitutional oaCGH analysis was performed on DNA purified from the skin biopsy obtained at the time for karyotyping. The oaCGH analysis was done against pooled male reference DNA and revealed a normal result, i.e. the observed microdeletions in the patient’s bone marrow cells were absent in his skin cells (Figure 3D-F). These results together with the normal karyotype from his skin biopsy show that the abnormal inv(2) karyotype with concurrent microdeletions were confined to the blood forming tissue and thereby acquired.

### Locus-specific FISH analysis

**Figure 4:**
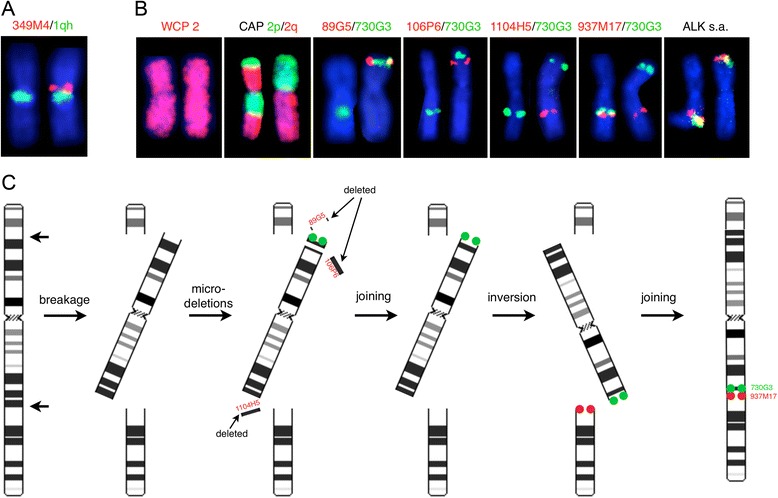
**FISH analyses for validation of array findings and a model of the complex chromosomal inversion of chromosome 2.** Partial karyograms of chromosome pair 1 **(Panel A)** and chromosome pair 2 **(Panel B)** with the aberrant chromosomes to the left showing FISH results after hybridization using the respective probes as indicated at the top. The relative positions of the directly labeled RP11-based BAC probes are indicated in Figure 3. Panel **C**. Schematic representation of the inversion on chromosome 2 with breakage points indicated by arrows, together with regions that are deleted and joined. Names of the deleted RP-11-based BAC probes are indicated in red, the moved RP11-730G3 probe is marked by filled green circles, and the RP11-937M17 is indicated by filled red circles. CAP: chromosome arms-specific painting probes; WCP: whole chromosome painting probes.

To validate the microdeletion in chromosome 1 band region p13.2 a locus-specific BAC-based custom-made probe was co-hybridized with a commercial probe of the heterochromatic region of chromosome 1q (Figure 4A). This experiment confirmed the 1p13.2 microdeletion, which was present in approximately 85% of the bone marrow cells.

To validate the microdeletions on chromosome 2 locus-specific FISH analyses were performed using several BAC-based custom-made probes, which pairwise were co-hybridized, and compared to dual color chromosome arms-specific painting probes of chromosome 2. These experiments showed: 1) the BAC-based probes RP11-89G5 (2p23.3), RP11-106P6 (2p22.1), and RP11-1104H5 (2q24.3) all exhibited mono-allelic deletions confirming the microdeletions as suggested by the oaCGH analysis; and 2) the microdeletions on chromosome 2 are located on the same derivative inverted homologue of chromosome 2 (Figure 4B). Counting 200 interphase nuclei using each of the BAC-probes showed that 87-90% of the interphase nuclei contained the microdeletions. Using the BAC-probe RP11-730G3 (2p23.3), located on the 12.8 Mb fragment situated between the two microdeletions at 2p23.3 and 2p22.1, and the BAC-probe RP11-937 M17 (2q31.1) it was confirmed that these probes are not in the deleted regions, as expected from the oaCGH analysis, and that the probes co-localize as a result of the inv(2) (Figure 4B). Analyzing 200 interphase nuclei with these probes the signal pattern 1F1R1G was observed in 85% of the nuclei and a signal pattern 2R2G in the remaining 15% of the cells confirming that inv(2) is present in approximately 85% of the cells.

The recurrent translocations t(2;5)(p23;q35)/NPM1-ALK and t(2;5)(p23;q35)/SQSSTM1-ALK, and inversion inv(2)(p23q35)/ATIC-ALK involving the ALK gene are well known rearrangements on chromosome 2 in various lymphoma types. To examine whether the *ALK* gene is involved in our patients inversion 2 rearrangement we used a commercially available dual color split apart probe surrounding the ALK gene and show that is not involved in any translocation or inversion although it is part of the chromosome 2 segment that is inverted (Figure 4B). Analyzing 200 interphase nuclei showed a 2F signal pattern in all examined cells.

## Discussion

We have characterized a pericentric inv(2)(p23.3q24.3) in a patient with chronic thrombocytopenia and moderate anemia. We identified a 141,8 Mb large inversion with four concurrent microdeletions which were acquired because cytogenetic examination and aCGH analysis of a skin biopsy were normal. The acquired chromosomal inv(2) is to the best of our knowledge novel as a search in Mitelman [13] and literature databases revealed no additional cases. In addition, a search in our local registry with more than 17,000 entries since 2001 was also without additional cases.

Inversions are considered balanced intrachromosomal rearrangements formed when a chromosome breaks in two places and the material between the two breakpoints reverses orientation before the breaks are sealed. Congenital pericentric inversions occur with an estimated frequency at 0.12-0.70% [14]. Some recurring inversions are considered normal variants including inversion in chromosomes 1, 9, and 16 involving the chromosomes heterochromatic region and in chromosomes 2, 3, 10 and in the Y-chromosome. These chromosomal variants are often familial and appear to segregate without deleterious effect [15].

Acquired inversions are also very rare. In hematological malignancies they are often variants of related translocations between the two chromosome homologues. The following recurring inversions with their translocation variants have been described in myeloid malignancies: inv(3)(q21q26)/t(3;3)(q21;q26) [16,17]; inv(11)(p15q22) [18]; and inv(16)(p13q22)/t(16;16)(p13;q22) [19], and as a result of these rearrangements the fusiongenes *PSMD2/MECOM, NUP98/DDX10, and CBFB/MYH11* are formed, respectively. No concurrent submicroscopic deletions have been described in these situations.

Recurrent inversion of chromosome 2 in hematological malignancies has only been described in patients with anaplastic large cell lymphoma resulting in the ATIC-ALK fusion gene as a result of inv(2)(p23q35) [20]. In other cancers, an inv(2)(p21p23)/*EML4-ALK*, has been described in non-small cell lung carcinoma [21]. In our patient we excluded that the *ALK* gene is involved in the pericentric inversion of chromosome 2 (Figure 4B).

As a result of the pericentric chromosome 2 inversion in our patient two of the detected microdeletions were at the inversion break point regions (2p23.3 and 2q24.3) and an additional in 2p22.1. A model of the inverted chromosome 2 is proposed (Figure 4C). This model summarizes the identified chromosomal events and should not be viewed as a model describing the order of chromosomal events. The complex rearrangement of chromosome 2 results from at least six DNA double-strand breaks (DSBs) giving rise to seven chromosome 2 fragments of different sizes. Three of the fragments were deleted as identified by the BAC-probes RP11-89G5, RP11-106P6 and RP11-1104H5 while a 12.8 Mb fragment (2p23.3p22.1) harboring the BAC-probe RP11-730G3 is either directly joined to the 128.95 Mb large chromosomal fragment encompassing the chromosomal bands 2p22.1 to 2q24.3 or it is inverted before it is joined to this fragment. The most likely interpretation is that the 12.8 Mb fragment is joined directly because the probes RP11-730G3 (2p23.3) and RP11-937 M17 (2q31.1) are co-localized in a similar way as the RP11-89G5 and the RP11-730G3, which are only a few kilobasepairs apart (Figure 4B). Finally, this now 141.8 Mb large centromere containing fragment is inverted and joined to the remaining two distal chromosomal fragments. *In silico* analysis of the involved chromosomal regions was performed by quering the UCSC database (http://genome.ucsc.edu) but gave no indications of possible fusion genes in our inv(2) patient disregarding possible long range genomic effects as a result of the rearrangements.

Chromosomal rearrangements are termed “complex” when a rearrangement involves more than two DNA strand breaks [22]. The mechanisms responsible for the development of complex chromosomal rearrangements remain unclear. According to a recently proposed model it is suggested that complex rearrangements might result from a single catastrophic event called “chromothripsis” as opposed to a progressive model with sequential accumulation of chromosomal aberrations [23]. In the chromothripsis model one or a few chromosomes or chromosomal regions shatter into tens to hundreds of pieces in a single event. Some of these pieces are haphazardly stitched together by the DNA repair machinery; while some pieces are lost resulting in severe localized complex chromosomal aberrations. Although neither of the two mechanisms seamlessly can explain the observed abnormalities of chromosome 2 in our patient it is most likely that the rearrangements occurred in a single event. It might be speculated that they result from DSBs formed from collapsed replication forks because over-initiation of replication has been identified in pre-cancerous cells that lead to collapse of replication forks [24,25]. Recently, it was uncovered that break-induced replication (BIR) is a DSB repair mechanism capable of recovering collapsed replication forks at the expense of producing genomic rearrangements with high frequencies, reviewed in [26]. BIR has also been suggested to be involved in chromothripsis.

A total of 104 RefSeq genes were deleted as a result of the submicroscopic deletions detected in our patient: 1) 38 RefSeq genes were deleted in 1p13.2; 2) 34 RefSeq genes were deleted in 2p23.3; 3) 25 RefSeq genes were deleted in 2p22.1; and 4) 7 RefSeq genes were deleted in 2q24.3 (Additional file 1: Table S1 for details).

In general, the products of the involved genes in the described microdeletions are mainly associated with signal transduction, cytoskeleton organization, transcription regulation and mRNA processing. There are, however, some genes in these regions that attract specific attention. Interestingly, the *DNMT3A* gene located in the deleted region at 2p23.3 is one of the major *de novo* methyltransferases in human cells. It is together with *DNMT3B* required for development during early embryogenesis and deletion of these genes is embryonic and perinatal lethal [27]. The *DNMT3A* gene has been implicated as one possible mediator of aberrant promotor methylation contributing to the hematopoietic disturbances in MDS [28]. In an *in vivo* model *DNMT3A* loss-of-function confers a preleukemic phenotype on murine hematopoietic stem cells [29]. The gene *ST7L* located at 1p13.2 is part of the observed deletions in our inv(2) patient. This gene has been suggested to be a novel tumor suppressor because its allelic loss has been implicated in various cancers including myeloid malignancy [30]. The *LRIG2* gene is another gene located in the observed 1p13.2 deletion in our patient. It is one of three human LRIG genes, *LRIG1, LRIG2*, and *LRIG3*, encoding single pass transmembrane proteins, reviewed in [31]. The *LRIG1* is a negative regulator of growth factor signaling functioning as a tumor suppressor in mice models whereas the function of *LRIG2* and *LRIG3* are less well defined.

These published findings cannot, however, be directly translated into determining the effects of the submicroscopic deletions in our inv(2) patient. One of the reasons for this is mainly because the patient has more than 100 genes affected by the microdeletions. This observation might relate to some of the common chromosomal abnormalities observed in MDS such as monosomy 5 or large interstitial deletions on one of the long arms of chromosome 5 [32]. In these instances a haploinsufficiency model has been proposed indicating that loss of a single allele of more than one gene on 5q may act in concert to alter hematopoiesis and disrupt normal differentiation [33]. Secondly, we have not examined the expression levels of the involved genes or determined whether the remaining alleles might have inactivating mutations.

Despite these facts our present findings might give some first clues to better understand how chromosomal and genomic aberrations are related to chronic thrombocytopenia with moderate anemia and no clear signs of MDS. Only few recurrent chromosomal aberrations associated with isolated congenital thrombocytopenia have been reported. In the thrombocytopenia-absent radius syndrome mutations in the *RBM8A* gene or a 1.35 Mb microdeletion in 1q21.1 have been linked [9,10], and in the Paris-Trousseau type thrombocytopenia with dysmegakaryopoiesis deletion at chromosome 11 band region q23 have been reported in several cases [34,35].

In cases where isolated thrombocytopenia evolves with additional cytopenias or in cases where these present together it is suggestive of possible underlying MDS or AML and examination of the bone marrow by morphology, flow cytometry and cytogenetic analyses is recommended [3].

## Conclusions

This study contributes to the identification of cytogenetic abnormalities related to thrombocytopenia with anemia in a patient with an indolent clinical course. It is the first report of chronic thrombocytopenia and anemia associated with acquired inv(2)(p23.3q24.3) as a sole cytogenetic abnormality together with concurrent submicroscopic deletions at 2p23.3, 2p22.1, 2q24.3 and 1p13.2. Investigation of similar cases will be necessary to clarify the clinical significance of the inv(2) and the observed microdeletions.

Even though more than 100 genes are located in the deleted regions it is possible to evaluate the clinical characteristics in order to further the understanding of their participation in the pathogenesis, and how genes may act in concert to alter hematopoiesis and disrupt normal differentiation.

This study also underlines the necessity to study hematological patients by different means to get a comprehensive picture of the genetic changes in connection with acquired disease. G-banding and molecular cytogenetics alone would not have identified the concurrent microdeletions, which were revealed by high-resolution aCGH analysis. By the aid of custom-made locus-specific FISH probes it was possible to validate the submicroscopic deletions as well as to determine more precisely the resultant inversion rearrangement which would not have been possible by aCGH analysis alone.

## Consents

Written informed consent was obtained from the patient. A copy of the written consent is available for review by the Editor-in-Chief of this journal.

## Additional file

Additional file 1: Table S1. Summary of genes located in the maximal region of submicroscopic deletions.

## Abbreviations

BAC: Bacterial artificial chromosomes
PHA: Phytohaemagglutinin
UCSC: University of California Santa Cruz
